# Determination of Phenolic Content in Different Barley Varieties and Corresponding Malts by Liquid Chromatography-diode Array Detection-Electrospray Ionization Tandem Mass Spectrometry

**DOI:** 10.3390/antiox4030563

**Published:** 2015-08-14

**Authors:** Daniel O. Carvalho, Andreia F. Curto, Luís F. Guido

**Affiliations:** REQUIMTE/LAQV—Departamento de Química e Bioquímica, Faculdade de Ciências, Universidade do Porto, Rua do Campo Alegre 687, 4169-007 Porto, Portugal; E-Mails: danielcarvalho@fc.up.pt (D.O.C.); andreiacurto@yahoo.com (A.F.C.)

**Keywords:** barley, malt, malting, polyphenols, phenolic acids

## Abstract

A simple and reliable method for the simultaneous determination of nine phenolic compounds in barley and malted barley was established, using liquid chromatography-diode array detection-electrospray ionization tandem mass spectrometry (HPLC-DAD-ESI-MS/MS). The phenolic compounds can be easily detected with both systems, despite significant differences in sensitivity. Concentrations approximately 180-fold lower could be achieved by mass spectrometry analysis compared to diode array detection, especially for the flavan-3-ols (+)-catechin and (−)-epicatechin, which have poor absorptivity in the UV region. Malt samples were characterized by higher phenolic content comparing to corresponding barley varieties, revealing a significant increase of the levels of (+)-catechin and (−)-epicatechin during the malting process. Moreover, the industrial malting is responsible for modification on the phenolic profile from barley to malt, namely on the synthesis or release of sinapinic acid and epicatechin. Accordingly, the selection of the malting parameters, as well as the barley variety plays an important role when considering the quality and antioxidant stability of beer.

## 1. Introduction

Barley is an abundant source of phenolic compounds and can be consider an excellent dietary matrix of natural antioxidants for disease prevention and health promotion. Additionally, barley phenolics are very important due to their influence in several stages of the brewing process and the overall beer stability (e.g., formation of haze, color, taste, filtration, foam stability and redox state) [1,2,3,4,5]*.* It is known that over 60% of the total polyphenolic content found in beer comes from barley [6].

Barley contains different classes of phenolic compounds, such as benzoic and cinnamic acid derivatives, proanthocyanidins, quinines, flavonols, chalcones, flavones, flavanones, and amino phenolic compounds. They can be found in a free, esterified or in an insoluble bound form and they are quantitatively distributed between different tissues of the grains [1,7,8,9,10,11,12,13,14,15]. Ferulic acid (4-hydroxy-3-methoxycinnamic acid) and *p*-coumaric acid (4-hydroxycinnamic acid) are the major low-molecular weight phenolic acids in barley grains, mainly found in the outer layers (husk, pericarp, testa, and aleurone), but also detected in endosperm. Other bound phenolic acids found in barley are vanillic, sinapinic, and *p*-hydroxybenzoic acids [2,9,14,16,17,18]. Barley grains also contain a range of flavan-3-ols from monomers ((+)-catechin and (−)-epicatechin), dimers (prodelphinidin B3 and procyanidin B3), and trimers (procyanidin C2), up to higher-molecular weight flavonoid-derived tannins [9,19,20]*.*

The malting process is responsible for modifications in the composition of barley, involving changes and degradation of endogenous phenolic compounds [5,9,15,16,21,22,23]. Some authors have demonstrated that the contents of phenolic compounds in malt are usually higher than in barley, but proportions of the different groups are nearly identical, suggesting a better extraction of flavonoids and phenolic acids in malt is possible after kilning [14,15,16]*.*

**Figure 1:**
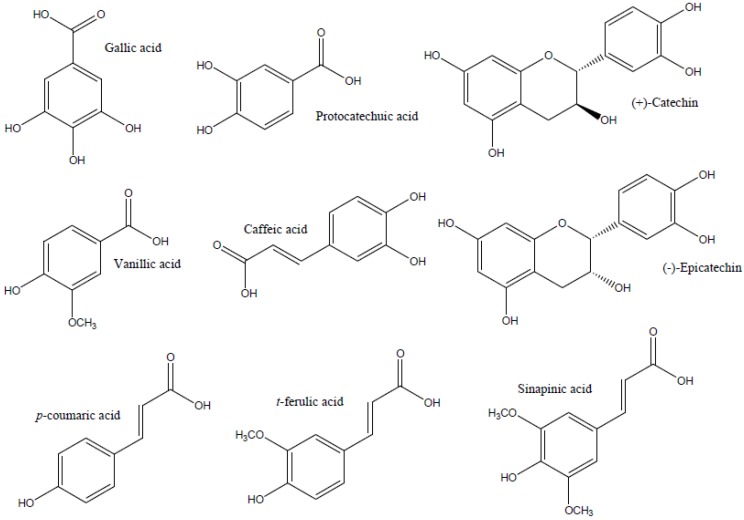
Structure of the phenolic compounds studied in this work.

The aim of this study was to establish a chromatographic method for identification and quantification of different phenolic compounds in barley and malt, as listed in Figure 1. An assay using liquid chromatography-diode array detection-electrospray ionization tandem mass spectrometry (HPLC-DAD-ESI-MS/MS) was established. The methodology was further applied in the determination of phenolic compounds in ten different barley varieties and their corresponding malts.

## 2. Materials and Methods

### 2.1. Chemicals

Acetic acid, boric acid, ortophosporic acid, hydrochloric acid, sulphuric acid, sodium chloride and sodium hydroxide were obtained from Prolabo (Fontenay-sous-Bois, France). 1,1-diphenyl-2-picryl hydrazil, sodium hidrogenosphosphate, sodium acetate trihydrate were purchased from Sigma-Aldrich (Saint Quentin Fallavier, France). Sodium dihydrogen phosphate monohydrate (p.a) was obtained from Merck (Fontenay-sous-Bois, France). Standards of (−)-epicatechin, (+)-catechin, *p*-coumaric acid, ferulic acid, caffeic acid, vanillic acid, protocatechuic acid, sinapic acid and gallic acid were purchased from Sigma (Germany). Acetonitrile, methanol, acetone and formic acid (Merck, Darmstadt, Germany) were of HPLC grade. Hydrogen chloride was purchased to Carlo ErbaReactifs (Val de Reuil, France) and was of analytical grade. High-purity water from a Simplicity 185 water purification system (Millipore Iberian, Spain) was used for all analyses and glassware washing.

### 2.2. Instrumentation

Two chromatographic systems were used. System 1 was a Jasco HPLC equipment (Jasco Corporation, Tokyo, Japan) composed by a LC-NETII/ADC interface, a Jasco quaternary gradient pump model PU-2089 Plus, a multiwavelenght detector model MD-1510 and a Jasco auto-sampler model AS-950. Data processing was made using Chrompass software version 1.8 (Jasco Corporation, Tokyo, Japan). The system 2 was a HPLC Finnigan-Thermo Electron Corporation (Thermo Electron Corporation, San Jose, CA, USA) consisting of a low-pressure quaternary pump and auto-sampler, both model Finnigan Surveyor plus. This system was equipped with two in-line detectors: a photodiode array and a mass spectrometric detector. The mass spectrometer consisted in a quadropole ion trap (Finnigan LCQ Deca XP Plus) equipped with an electrospray ionization (ESI) source. Data acquisition and processing was made using Xcalibur software version 1.4 (Thermo Electron Corporation, San Jose, CA, USA).

### 2.3. Chromatographic Conditions

For separation, a LiChroCart (Merck, Darmstadt, Germany) RP-C_18_ column (125 mm × 3.0 mm, 3 µm) connected to a guard column (LiChroCart RP-C_18_ 4.0 mm × 4.0 mm, 5 µm) was used with a mobile phase consisting of an aqueous formic acid solution (0.1%; A) and methanol (B). The flow rate was 0.3 mL/min and started with a mixture of 90% A and 10% B, raised to 60% B and 40% A during 75 min, turned back to 90% A in 5 min and kept for 15 min for reconditioning before the next injection. A total of 20 µL was injected into the column kept at room temperature.

Mass spectrometer conditions (only for system 2): negative mode, capillary temperature, 275 °C; source voltage, 4.5 kV; capillary voltage, −5.0 V; sheath gas (N_2_) flow at 80 arbitrary units and auxiliary gas (N_2_) flow rate at 10 arbitrary units. During the chromatographic run, mass spectra of the eluate were recorded from m/z 100 to m/z 1000 and tandem mass spectrometry experiments were carried out. For quantification the SIM mode was used.

### 2.4. Standard Solutions and Calibration Curves

For quantification purposes, the external standard calibration was used. Peak areas from the HPLC chromatogram were plotted against the known concentrations of standards at varying concentrations. Equations generated by linear regression (using Excel software) were used to establish concentrations of the phenolic compounds.

About 100 mg of each standard, accurately weighted, was dissolved in 100.0 mL of methanol (using a 100.0 mL volumetric flask) to obtain stock solutions. The actual concentration was calculated and recorded for ulterior dilutions. The stock solutions were kept in the dark at −20 °C.

The stock solutions were checked periodically (once a week) for degradation. The standards were stable for at least a month. Since each stock solution was prepared freshly on a monthly basis, no further investigation was undertaken on this subject.

For calibration curves, each stock solution was diluted with water to obtain the concentration sequence. The linear range and the equations of linear regression were obtained through successive injections of standards of varying concentrations (concentration range between 1 mg/L and 100 mg/L, except for (+)-catechin and (−)-epicatechin where the range was between 5 mg/L and 250 mg/L). Mean areas (*n* = 4) generated from the standard solutions were plotted against concentration to establish calibration equations (using Excel software).

### 2.5. Elaboration of the Library of UV-Vis Spectra of the Standards

Each standard was injected separately in HPLC system 1 in order to get the spectrum for each compound in the condition of analysis. The concentration of each standard injected was 50 mg/L. For the tuning of the mass detector (direct infusion experiments), the standards were directly injected into the mass detector. Standards were diluted from the stock solutions with methanol to a final concentration of 10 mg/L. Each standard was injected separately in the MS spectrometer in order to obtain both the best conditions in the ionization source and the fragmentation patterns.

### 2.6. Instrumental Detection Limits

For the evaluation of detection limits (of each instrument), all the standards were dissolved separately in mobile phase (water:methanol 90:10 v:v) (cf. analytical conditions for further details) to a final concentration of 100.0 mg/L (except for (+)-catechin, (-)-epicatechin where the final concentration was 250 mg/L). Each stock solution was diluted with mobile phase and standards with a concentration of 50.0, 25.0, 10.0, and 5.0 mg/L were obtained.

### 2.7. Repeatability (Instrumental)

The standard solutions of 10 mg/L (25 mg /L for (+)-catechin and (−)-epicatechin), were used to achieve repeatability testing for intraday and interday (*n* = 4). The data used to calculate relative standard deviation (RSD) percent of inter-day repeatability was the mean value of three injections in succession.

### 2.8. Barley and Malt Extract Preparation

Thirty grams of finely powdered malt and barley samples (Esterel, Alexis, Tocada, Arturio, Frilox, Séduction, Class, Propice, Regalia and Scarlett) were extracted twice with 100 mL of methanol and centrifuged (10,000× *g*, 10 min, room temperature). Both supernatants were collected and filtered (Whatman). The samples were then evaporated to dryness under vacuum at 45 °C. The residue was dissolved in 6.0 mL of ultra-pure water and filtered with a polytetrafluoroethylene (PTFE) filter (0.45 μm, Machery-Nagel) (VWR, Darmstadt, Germany) before HPLC analysis.

## 3. Results

### 3.1. Chromatographic Separation of Phenolic Compounds

**Figure 2:**
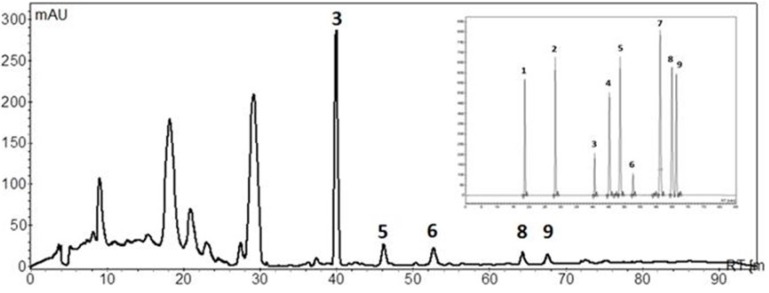
Example of a chromatogram obtained for a sample of malted barley extract. A chromatographic peak profile obtained for the phenolic standards is shown in the insert. The concentration of phenolic standards was approximately 50 mg/L. The chromatogram was obtained for the maximum absorbing wavelength at each time point. 1: gallic acid; 2: protocatechuic acid; 3: (+)-catechin; 4: vanillic acid; 5: caffeic acid; 6: (−)-epicatechin; 7: *p*-coumaric acid; 8: ferulic acid; 9: sinapinic acid.

In a first approach, the UV cut-offs of the different solvents used for HPLC separation of polyphenols were established. The UV cut-off defines the UV wavelength at which the absorbance of the solvent in a 1 cm cell (*versus* air as reference) is equal to unity. The UV cut-offs of the most common solvents used in RP-HPLC are well established and documented [24]. Solvents with high cut-offs, such as acetone, ethyl acetate and DMSO are not suitable for analysis at low wavelengths, such as 250 nm. A solvent with a UV cut-off higher than the working wavelength used for an analysis generates such a high background absorbance that it is excluded from further consideration. Accordingly, acetonitrile, methanol and THF (and of course water) were selected from all the possibilities tested. The chromatographic separation of polyphenols, in the system 2, was performed by using a binary mixture of acidified water and methanol. A chromatogram demonstrating the separation of standards is shown in Figure 2, demonstrating a good resolution for all the phenolic standards. The concentration of the mixed standard solution was 50 mg/L for each polyphenol.

Spectral peak matching and purity calculation were performed using the Chrompass software version 1.8 based on a correlation algorithm, whereas a correlation matching factor between the sample and reference spectra superior to 990 means that peaks are similar; between 900 and 990 represents some similarity and inferior to 900 indicates differences. Sample peaks were identified assuming a rejection criteria value of 900.

### 3.2. MS and DAD Analyses of Phenolic Compounds

**Figure 3:**
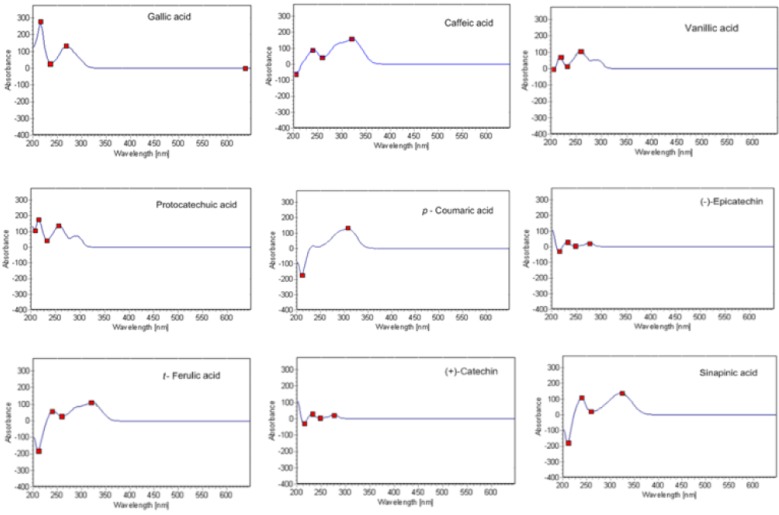
UV spectra recorded for each phenolic compound.

Every standard available was injected separately in HPLC system 2 using the conditions described in “Material and Methods” in order to get its UV spectrum. The UV spectrum obtained on-line after HPLC separation of each phenolic compound may be influenced by the nature of the solvent, which varies along the chromatographic run. The recorded spectrum for each compound is presented in Figure 3. The conditions were selected as follows: 250 nm for vanillic acid, 275 nm for gallic and syringic acids, (+)-catechin and (−)-epicatechin and 320 nm forcaffeic, sinapinic, *p*-coumaric and ferulic acids.

Table 1 lists the UV bands of the phenolic standards, as well as their pseudo-molecular ions using ESI-MS in negative ionization mode. Positive and negative ion modes were tried for the phenolic standards, and the results suggested that negative mode was more sensitive. ESI source gave the deprotonated molecular ion ([M-H]^−^) as the base peak. The pseudo-molecular ion was then selected for CID fragmentation to produce MS^2^ spectra (collision energy ranged from 25% to 45%).

**Table 1:** Pseudo-molecular ions and product ions of phenolic compounds, as well as their UV characteristics bands.

Standard	[M-H]^−^ m/z (MS)	Fragments m/z (MS^2^) (intensity)	UV band (nm)
Gallic acid	169	125 (100)	272
Protocatechuic acid	153	109 (100)	260 (max), 294
(+)-Catechin	289	205 (30), 245 (100), 179 (15)	280
Vanillic acid	167	123 (100)	260 (max), 294
Caffeic acid	179	135 (100)	324 (max), 296
(−)-Epicatechin	289	205 (30), 245 (100), 179 (20)	280
*p*-Coumaric acid	163	119 (100)	310
*t*-Ferulic acid	193	134 (20), 149 (100), 175 (40)	324 (max), 296
Sinapinic acid	223	208 (100), 179 (40), 164 (30)	324

In the negative ion mode, hydroxybenzoic acids produce the deprotonated molecule and a [M-H]^−^fragment ion via loss of a CO_2_ group from the carboxylic acid moiety. The UV spectra of the hydroxybenzoic acids are quite relevant to their chemical structures. Single absorption peaks appear in the UV spectrum of gallic acid (which has a symmetrical structure), whereas in the case of phenol, such as protocatechuic acid and vanillic acid, which have non-symmetrical chemical structures, two absorption peaks were noted in the corresponding UV spectra (Figure 3). Like the hydroxybenzoic acids, hydroxycinnamic acids, such as caffeic acid, also produced a deprotonated molecule ([M-H]^−^) and lost a CO_2_ group (from the carboxylic acid function) in the negative ion mode ([M-H-44]^−^). Ferulic acid and sinapinic acid showed the neutral loss of a water molecule, providing a ([M-H-18]^−^) product ion at m/z 175 and m/z 205, respectively. Sinapinic acid and *p*-coumaric acid, which have a plane of symmetry with respect to the surface of the molecule (both have planar geometry, [25]), showed a single absorption peak in the UV spectra. On the other hand, caffeic and ferulic acids, with no plane of symmetry, had a major absorption peak and along with a shoulder absorption under these conditions. The reason for this discrepancy may be the substitution of hydroxyl or methoxyl groups of the cinnamic-type, which caused hypsochromic shifts.

Regarding the flavan-3-ols, (+)-catechin ([M-H]^−^ m/z 289) yielded product ions at m/z 245, 179, 205. The electrospray ionization (−) tandem mass spectrometry (ESI (−)-MS/MS) fragmentation pattern of catechin has been already described by Callemien and Collin [26]. The isomer (−)-epicatechin gave the same product ions, as the stereoisomers could not be distinguished by mass spectrometry. The product ion at m/z 245 ([M-H-44]^−^) in both flavan-3-ols was produced by the loss of a (CH)_2_OH group; the fragment at m/z 205 is probably due to a loss of flavonoid A-ring. The fragment at m/z 179 may be due to the loss of the B-ring from the flavonoid. The UV absorption spectra of both flavan-3-ols showed a single peak at wavelength of 280 nm (Figure 3).

### 3.3. Analytical Detection Limits

Stock solutions of the available standards were prepared, diluted and calibration curves were developed. A linear relationship between concentration and peak area ratio was confirmed from the slope of the linear calibration and acceptability of linearity was to be judged from a correlation coefficient (*R*^2^) being greater than 0.99.

The calibration curves obtained for each compound as well as the limit of detection (LOD) and limit of quantitation (LOQ) for each compound were calculated (Table 2). LOD and LOQ were determined based on the standard deviation of the response and the slope from the calibration curve for each compound, according to the ICH (International Conference on Harmonization) recommendations. LODs and LOQs were estimated as 3.3 and 10 times of the standard deviation of the blank/slope ratio of the calibration curve, respectively. The standard deviation of *y*-intercepts of regression lines were used as the standard deviation of the blank. In order to compare the sensitivity of the DAD and MS analyses, the same calibration curve was made using system 2. The results obtained are presented in Table 3.

**Table 2:** Analytical parameters obtained for the HPLC-DAD analysis.

Standard	Range (mg/L)	Slope	Intercept	Correlation Coefficient (*R*^2^)	Reproducibility (%)	Analytical Limits (mg/L)	
						LOD	LOQ
(+)-Catechin	5–250	0.34	0.65	0.99	2.5	2.11	6.35
Vanillic acid	1–100	1.61	−2.98	0.99	1.2	0.50	1.50
Caffeic acid	1–100	2.85	−4.94	0.99	0.9	0.31	0.93
(−)-Epicatechin	5–250	0.34	−0.63	0.99	2.9	2.07	6.21
*p*-Coumaric acid	1–100	2.91	−5.12	0.99	0.5	0.27	0.80
*t*-Ferulic acid	1–100	2.82	−6.11	0.99	0.7	0.30	0.90
Sinapinic acid	1–100	2.55	−6.50	0.99	1.0	0.32	0.95

### 3.4. Determination of Phenolic Compounds in Barley and Corresponding Malt

The content of single polyphenols were determined in 10 different varieties of barley and corresponding malts (Table 4 and Table 5, respectively).In barley samples the main phenolics identified were (+)-catechin, vanillic acid, caffeic acid, *p*-coumaric acid and ferulic acid. In malt, vanillic acid was not identified. However, epicatechin, sinapinic acid and traces of cinnamic acid were characteristic of malted barley samples. Catechin was the phenolic in higher concentrations both in barley and malt, varying from 20.8 to 70.4 mg/kg DW and 64 to 604 mg/kg DW, respectively.

**Table 3:** Comparison between MS and DAD detector.

Standard	Slope UV	Slope MS	Ratio MS/UV
Gallic acid	1.3 × 10^6^	3.0 × 10^7^	24
Protocatechuic acid	1.7 × 10^6^	3.7 × 10^7^	21
(+)-Catechin	4.3 × 10^5^	7.4 × 10^7^	174
Vanillic acid	2.0 × 10^6^	4.3 × 10^7^	21
Caffeic acid	2.5 × 10^6^	8.9 × 10^7^	36
(−)-Epicatechin	4.3 × 10^5^	8.0 × 10^7^	186
*p*-coumaric acid	2.8 × 10^6^	7.9 × 10^7^	28
*t*-Ferulic acid	2.8 × 10^6^	8.6 × 10^7^	32
Sinapinic acid	2.5 × 10^6^	1.4 × 10^8^	53

**Table 4:** Phenolic content of different barley varieties.

Phenolic Compound (mg/kg DW)	Esterel	Alexis	Regalia	Propice	Arturio	Séduction	Tocada	Class	Frilox	Scarlett
Gallic acid	ND	ND	ND	ND	ND	ND	ND	ND	ND	ND
Protocatechuic acid	ND	ND	ND	ND	ND	ND	ND	ND	ND	ND
(+)-catechin	70.4	32.0	20.8	37.2	59.3	69.8	52.2	47.6	42.5	46.3
Vanillic acid	ND	ND	ND	ND	ND	1.4	ND	0.3	ND	ND
Caffeic acid	1.1	3.9	2.6	1.3	0.9	0.8	1.1	2.9	ND	ND
(−)-epicatechin	ND	ND	ND	ND	ND	ND	ND	ND	ND	ND
*p*-coumaric acid	1.3	1.1	0.4	2.1	1.4	2.1	0.1	1.1	ND	ND
Ferulic acid	2.1	4.2	1.4	4.3	1.8	3.3	2.6	3.0	0.1	0.1
Sinapinic acid	ND	ND	ND	ND	ND	ND	ND	ND	ND	ND

DW: dry weight; ND: not detected.

**Table 5:** Phenolic content of different malted barley varieties.

Phenolic Compound (mg/kg DW)	Esterel	Alexis	Regalia	Propice	Arturio	Séduction	Tocada	Class	Frilox	Scarlett
Gallic acid	ND	ND	ND	ND	ND	ND	ND	ND	ND	ND
Protocatechuic acid	ND	ND	ND	ND	ND	ND	ND	ND	ND	ND
(+)-Catechin	119	64	96	221	343	428	75	65	243	604
Vanillic acid	ND	ND	ND	ND	ND	ND	ND	ND	ND	ND
Caffeic acid	2.0	4.1	2.0	16.5	41.3	1.0	4.0	3.2	18.8	7.4
(−)-Epicatechin	6.4	11.7	7.7	5.4	9.7	ND	ND	ND	5.1	5.4
*p*-coumaric acid	ND	ND	ND	ND	ND	ND	ND	ND	ND	ND
Ferulic acid	2.1	2.5	2.1	1.9	1.5	2.1	1.8	1.7	2.2	2.3
Sinapinic acid	0.7	0.9	0.6	1.2	2.0	1.0	1.0	1.1	1.2	0.9

DW: dry weight; ND: not detected.

## 4. Discussion

### 4.1. Chromatographic Separation of Phenolic Compounds

The chemical nature of phenolic compounds must be taken into account when designing a specific method for their separation. The structures of the phenolic compounds in study are shown in Figure 1. The analyzed phenolic compounds can be divided into three main categories: benzoic acid derivatives (gallic acid, protocatechuic acid and vanillic acid), cinnamic acid derivatives (caffeic acid, coumaric acid, ferulic acid and sinapic acid) and flavan-3-ols ((+)-catechin and (−)-epicatechin).

The basis for the analyte retention on chromatographic separations using reversed phase columns, such as that used in this work, is the competitive interaction of the analyte and eluent components with the hydrophobic stationary phase. The polarity of phenolic compounds depends mostly on the number of hydroxyl/methoxyl groups in the aromatic ring. The stronger the interaction, the longer retention time will be observed. The analyte nature and its appearance (e.g., ionization state) affect the retention mechanism. Therefore, the eluent pH will greatly affect separation, namely by disturbing the ionization equilibrium of the analyte. The elution mode used in system 2 was a binary mixture of solvents composed by acidified water and an organic modifier, in this case methanol. Other solvents can be used instead of methanol. The most common is acetonitrile, but other solvents as tetrahydrofuran [27] or ethyl acetate [28] have also been reported to be successful. The acidification of water is necessary to suppress the ionization of the phenolic hydroxyl groups, thus obtaining sharper peaks and minimizing peak tailing. Typically, sulfuric, phosphoric, formic, acetic, and trifluoroacetic acids are the most common additives in the mobile phase. Considering the pKa of formic acid (3.75) and acetic acid (4.76), these were the first choices for the experiments. Meanwhile, in view of the potential for detection of the analytes by LC-ESI-MS in negative mode, formic acid used at a concentration of 0.1% resulted in symmetric peaks. Higher concentrations of acid were not necessary and were also avoided for two reasons: firstly, HPLC-MS is very sensitive to high acid concentrations which may induce ion suppression and second, the C18 column has a working pH between 2 and 12. Secondly, lowering the pH may induce chemical damage to the column and reduce its shelf-life.

Flavan-3-ols, like the epimers (+)-catechin and (−)-epicatechinpresent more problems in peak assignment. The basic unit of flavan-3-ols differs not only on the hydroxylation pattern but also on the stereochemistry of the asymmetric carbons. The spectral characteristics of flava-3-ols do not allow their easy detection and identification on the chromatograms. These compounds exhibit maximum absorption at non-specific wavelengths (270–290 nm), at which many other phenolic compounds also absorb, thus not allowing their selective detection. However, they are characterized for having very different molecular polarity, due to their different dipole moments, which is reflected in their retention times in the chromatogram (Figure 2). The use of standards is therefore necessary for the correct identification and quantification.

### 4.2. Analytical Detection Limit

The HPLC-DAD analyses showed that the flavan-3-ols had a very low response factor, making the quantification of these compounds rather difficult when they are in low concentration. In contrast, the response factor for the phenolic acids was quite good.

The increase in sensitivity in the MS analysis (SIM mode) confirms the ability of mass spectrometers coupled to liquid chromatography for quantitative determinations of phenolic compounds. The highest increase was obtained for (+)-catechin and (−)-epicatechin, mainly because these two compounds have, as mentioned earlier, low extinction coefficients in the UV region. Despite the increment in sensitivity using the MS, HPLC-DAD is still the most common method to determine phenolic compounds.

Mass spectrometry analyses could detect concentrations around180-fold lower than DAD, especially for (+)-catechin and (−)-epicatechin, which exhibit poor absorptivity in the UV region.

### 4.3. Determination of Polyphenols in Malt and Corresponding Malt

Flavan-3-ols were the major free phenolics identified in barley and malt. (+)-Catechin is the most abundant phenolic compound both for barley and malt. (−)-Epicatechin is not present in barley, as it has been reported that the content of epicatechin significantly increases during malting by partial conversion from catechin [20]. Likewise, sinapinic acid was not detected in the barley samples, however it was found in malt at low concentrations (between 0.6 mg/kg DW and 2.0 mg/kg DW).

Catechin and ferulic acid have been described as the most abundant phenolics identified, respectively, in free and bound fractions [9,15,19,29,30]. Our results are in agreement with this, since catechin was found in higher concentrations in barley and malted barley, comparing to the other phenolic analyzed compounds (Table 3 and Table 4). Ferulic acid levels were found in the range 0.1–4.3 mg/kg DW for barley and in the range 1.5–2.5 mg/kg DW for malt. The extraction with methanol does not allow the extraction of bound phenolics and only encompass the free and water soluble fractions [9]. For this reason, the ferulic acid levels found were affected since they are mainly present in the bound fraction [31].

The observed increase of catechin during the malting process can be attributed to the release of free phenolics by enzymes synthesized in the germination of the grain. Friedrich and Galensa [32] reported the existence of a catechin glucoside in barley that could be released during malting directly, by activated or developed enzymes. Accordingly, the amount of this glucoside significantly increases during malting. Samaras *et al*. [5] have showed similar results for catechin, ferulic and coumaric acids. In fact, barley germination was found to be responsible for an increase of the total phenolic content [33]. However, Goupy *et al*. [16] have showed a decrease in catechin content as well as in its dimmers and trimers. Malting is responsible for a large decrease of the levels of catechin, prodelphinidin B3, procyanidin B3 and ferulic acid from barley [5,9,15,16,21]. Additionally, a decrease of bound phenolics and an increase of soluble esterified fraction was observed during malting [9]. These changes were attributed to the enzymatic release of bound phenolic compounds of barley and with glycosylation reactions during malting, leading to higher levels of free phenolic acids and easier extractability due to changes in the matrix in the early kilning [5,9,22,23].

## 5. Conclusions

In this work, a separation and quantification method for phenolic compounds using HPLC-DAD-SI-MS/MS methodology is described. The phenolic compounds can be easily detected with both systems, despite significant differences in sensitivity. Mass spectrometry analysis can achieve concentrations approximately 180 times lower than UV-vis detectors, especially with compounds like (+)-catechin and (−)-epicatechin which have poor absorptivity in the UV region.

Malt samples were characterized by higher phenolic content comparing to corresponding barley, revealing a significant increase of the levels of flavan-3-ols catechin and epicatechin during the malting process. Moreover, the industrial malting is responsible for modification on the phenolic profile from barley to malt, namely on the synthesis of sinapinic acid and epicatechin. Accordingly, the selection of the malting parameters as well as the barley variety seems to be important factors when considering the quality and antioxidant stability of beer.
